# Cutaneous Squamous Cell Carcinoma in the Sacral Region With Regression Attributed to an Abscopal Effect Induced by Nivolumab and Radiotherapy: A Case Report

**DOI:** 10.1155/crdm/4943291

**Published:** 2026-07-01

**Authors:** Tomomichi Shimizu, Akio Kondoh, Hinako Chiba, Shinpei Hamasaki, Sho Arai, Tomomi Inomata, Ayako Hirota, Fumikazu Yamazaki, Tomotaka Mabuchi

**Affiliations:** ^1^ Department of Dermatology, Tokai University School of Medicine, Isehara, Kanagawa, Japan, u-tokai.ac.jp

**Keywords:** abscopal effect, cutaneous squamous cell carcinoma, immune checkpoint inhibitor

## Abstract

A 78‐year‐old Japanese man with a scar‐like sacral lesion was referred to our hospital due to a red tumor that had developed in the region. Pathological examination of a partial skin biopsy revealed cutaneous SCC. The SCC was regarded as “a Marjolin ulcer” because the tumor developed on a chronic scar. After his first surgery, the tumor had recurred in his sacral region. He underwent a second surgical procedure; however, metastases developed in his inguinal lymph nodes. He was started on nivolumab therapy for four cycles; however, he developed local recurrence in the sacral region. He subsequently underwent local radiotherapy to the recurrent sacral lesion. After completion of local RT, nivolumab was continued for two additional cycles. Two months after his RT, his lymph node metastases surprisingly regressed, although direct radiation was not administered to his inguinal lymph nodes. The present case represents the first reported instance of cutaneous SCC (Marjolin ulcer) in the sacral region treated with nivolumab and RT in which an abscopal effect was observed.

## 1. Introduction

The abscopal effect, a phenomenon in which localized radiation therapy at one site may lead to the regression of tumors at distant, nonirradiated sites, was first described in 1953 [1, 2]. In recent years, treatments for advanced‐stage cutaneous malignant tumors, such as immune checkpoint inhibitors (ICIs), have increased. The objective response rate and disease control rate of ICIs in advanced cutaneous squamous cell carcinoma (SCC) were reported as 47.2% and 64.4% [3].

The etiology of cutaneous SCC involves tumor‐induced lymphangiogenesis and upregulation of vascular endothelial growth factor‐C/D (VEGF‐C/D), which facilitates entry into the lymphatic system and promotes access to regional lymph nodes. Tumor‐stroma crosstalk (angiogenic signaling such as VEGF), recruitment of immunosuppressive cells, MHC‐I downregulation, production of immunosuppressive cytokines (IL‐10 and TGF‐β), and progression disease (PD)‐L1 expression collectively contribute to immune escape and shapes response to ICIs. The requirement for a pre‐activated immune microenvironment for effective checkpoint blockade is supported by evidence that baseline PD‐L1 expression and pre‐existing tumor‐infiltrating lymphocytes (TILs) correlate with higher response rates [4].

Additionally, Marjolin’s ulcer is a subtype of SCC and is associated with a poorer prognosis and a higher recurrence rate than non‐Marjolin SCC [5]. Treatment options for cases with postoperative recurrence or lymph node metastasis include radiotherapy or ICIs. Furthermore, combining these modalities may improve treatment outcomes through the abscopal effect.

Here, we describe a case of cutaneous SCC in which the abscopal effect was observed.

## 2. Case Presentation

**FIGURE 1:**
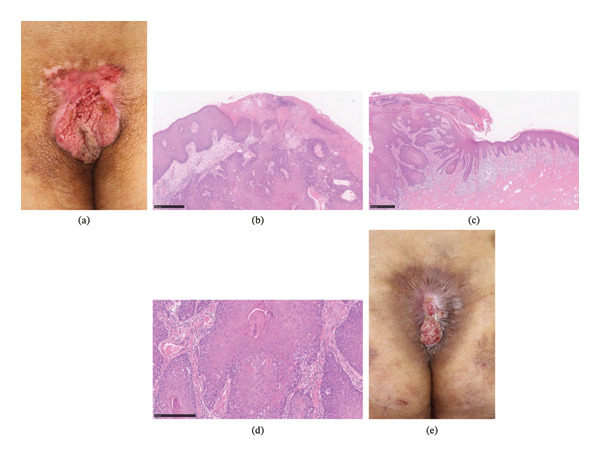
(a) A red tumor in continuity with a scar‐like plaque in his sacral region. (b) Hematoxylin‐eosin‐stained specimen showing asymmetry and partial ulceration. (c, d) Continuous proliferation of well‐differentiated and invasive tumor cells originating from the epidermis. (e) Recurrence of the tumor in his sacral region.

**FIGURE 2:**
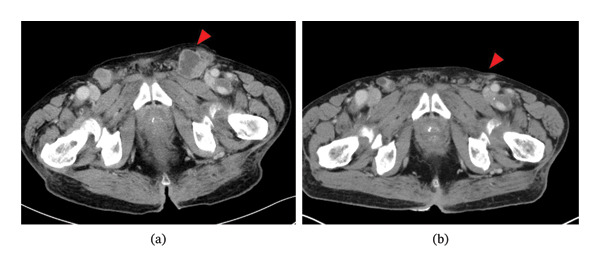
(a) Lymph node metastases in the inguinal and external iliac regions. (b) Regression of the lymph node metastases.

A 78‐year‐old Japanese man with a scar‐like sacral lesion was referred to our hospital due to a red tumor that had developed in the region (Figure 1a). The serum SCC antigen level was 2.2 ng/mL, and the lactate dehydrogenase (LDH) level was 210 U/L. Pathological findings of a partial skin biopsy showed cutaneous SCC. A wide local excision with a 1‐cm surgical margin was performed. Histopathological examination showed continuous proliferation of well‐differentiated and invasive tumor cells originating from the epidermis (Figure 1b–d). The depth of invasion was 20 mm, with infiltration into the subcutaneous tissue at the deep margin; no perineural invasion (PNI) was observed, whereas lymphovascular invasion (LVI) was present. An increase in collagen fibers was observed on the dorsal side of the resected specimen, which had been clinically considered to be a scar (Figure 1a, c). One month after his first surgery, the tumor had recurred in his sacral region (Figure 1e). He underwent a second surgical procedure, and the pathological findings confirmed complete tumor resection with negative margins. However, metastases developed in his inguinal lymph nodes (Figure 2a). Lymph node metastases were identified based on short axis, heterogeneity, and rim enhancement, indicative of central necrosis [6, 7]. The inguinal lymph node measured 50 mm in the long axis and 38 mm in the short axis, with findings indicative of central necrosis. The serum SCC antigen level was 9.4 ng/mL, and the LDH level was 192 U/L. He was started on nivolumab therapy (240 mg every two weeks) for four cycles; however, he developed local recurrence in the sacral region. He subsequently underwent local radiotherapy (RT: 30 Gy in 10 fractions) to the recurrent sacral lesion. After completion of local RT, nivolumab was continued for two additional cycles until the next CT scan assessment. Two months after his RT, his lymph node metastases surprisingly regressed, although direct radiation was not administered to his inguinal lymph nodes (Figure 2b). The inguinal lymph node measured 8 mm in the long axis and 5 mm in the short axis, and the sacral tumor had regressed. No new lesions were detected on imaging, and the overall response was evaluated as a complete response (CR) according to RECIST version 1.1 for this nodal lesion. The serum SCC antigen level was 2.2 ng/mL, and the LDH level was 185 U/L. He has continued treatment with nivolumab since then. After starting nivolumab therapy, he received 24 cycles of nivolumab over 18 months, and CR was maintained. He experienced no immune‐related adverse events (irAEs) from the nivolumab and no significant radiation dermatitis from the localized RT.

## 3. Discussion

RT is capable of altering the tumor microenvironment by inducing immunogenic tumor cell death and promoting the release of tumor‐associated antigens [8]. These antigens are processed by antigen‐presenting cells, leading to T‐cell activation and expansion [8]. Activated effector T‐cells can subsequently migrate to distant metastatic sites and exert antitumor effects [8]. However, activation of the PD‐1/PD‐L1 pathway may attenuate these immune responses [8]. Therefore, ICIs may enhance radiotherapy‐induced antitumor immunity by sustaining T‐cell activity through blockade of this pathway. It has been suggested that combining radiation therapy with ICIs targeting CTLA‐4 and PD‐1/PD‐L1 may incidence the increase of the abscopal effect in patients with metastatic or advanced cutaneous cancers [9].

It has been reported that there are very few reports demonstrating the abscopal effect with RT and ICIs in cutaneous SCC [10]. According to previous reports [10], 4 patients with advanced, inoperable disease were reported. All patients had cutaneous SCC originating in the head and neck region. The initial treatment was surgery, and all patients subsequently developed advanced disease. They received pembrolizumab at 2 mg/kg every 3 weeks, and radiotherapy was added in all cases. Case 1 was an 83‐year‐old man with cutaneous SCC originating in the vertex and presenting with unresectable in‐transit cutaneous metastases, whose disease showed PD after 4 cycles of pembrolizumab. Case 2 was an 82‐year‐old man with cutaneous SCC originating in the right ear and presenting with rapid enlargement of right cervical lymph nodes, whose disease showed CR after 18 cycles of pembrolizumab. Case 3 was a 77‐year‐old man with cutaneous SCC originating in the vertex. He presented with unresectable in‐transit cutaneous metastases and right basal‐cervical lymph node metastases. The disease had progressed after treatment with carboplatin, 5‐fluorouracil, and cetuximab, followed by paclitaxel. After 2 cycles of pembrolizumab, the disease showed PD. Case 4 was a 94‐year‐old man with cutaneous SCC originating in the right frontoparietal region and presenting with bone and leptomeningeal involvement, whose disease showed CR after 16 cycles of pembrolizumab. These report cases suggested that pembrolizumab with concurrent RT was well tolerated, and two patients achieved CR. In contrast to the previous report, nivolumab was used in our case; inguinal lymph node metastases notably regressed 2 months after RT. Additionally, unlike the previous report, our case of cutaneous SCC was a Marjolin ulcer, and RT was administered only to the primary lesion in the sacral region; this favorable outcome was considered to reflect an enhanced abscopal effect induced by the combination of RT and nivolumab. However, the development of inguinal lymph node metastases may have represented a delayed ICI response or pseudoprogression, as our case had received 4 cycles of nivolumab. Diagnosis and treatment response were assessed radiologically according to RECIST Version 1.1 and were not confirmed histologically by biopsy or fine‐needle aspiration.

In the present case, the SCC was regarded as a Marjolin ulcer arising from a chronic scar. The term “Marjolin ulcer,” originally described by the French surgeon Jean Nicolas Marjolin, was initially used to denote SCC arising in burn scars, but it has since been broadened to include various skin malignancies developing in previously injured or chronically damaged tissue [5]. Marjolin ulcers are generally associated with a poorer prognosis than de novo tumors of comparable histological type [5]. The present case represents the first reported instance of cutaneous SCC (Marjolin ulcer) in the sacral region treated with nivolumab and RT in which an abscopal effect was observed.

In conclusion, to our best knowledge, this case is the first report of a Marjolin ulcer with ICI and RT showing the abscopal effect. A previous report has suggested that combination therapy with RT and ICIs is effective for cutaneous SCC [9]. In our advanced case with recurrence of the primary lesion and inguinal lymph node metastases, an abscopal effect and synergistic effects were expected.

## Funding

No external funding was received for this case report.

## Consent

Written informed consent was obtained from the patient in accordance with the journal’s patient consent policy. The corresponding author will retain the original consent form and provide it to the publisher upon requested.

## Conflicts of Interest

The authors declare no conflicts of interest.

## Data Availability

The data that support the findings of this study are available from the corresponding author upon reasonable request.
